# Supplementary figures and images for: KCTD19 and its associated protein ZFP541 are independently essential for meiosis in male mice
Source: PLoS Genet. 2021 May 7;17(5):e1009412. doi: 10.1371/journal.pgen.1009412 (PMC8104389; doi:10.1371/journal.pgen.1009412)

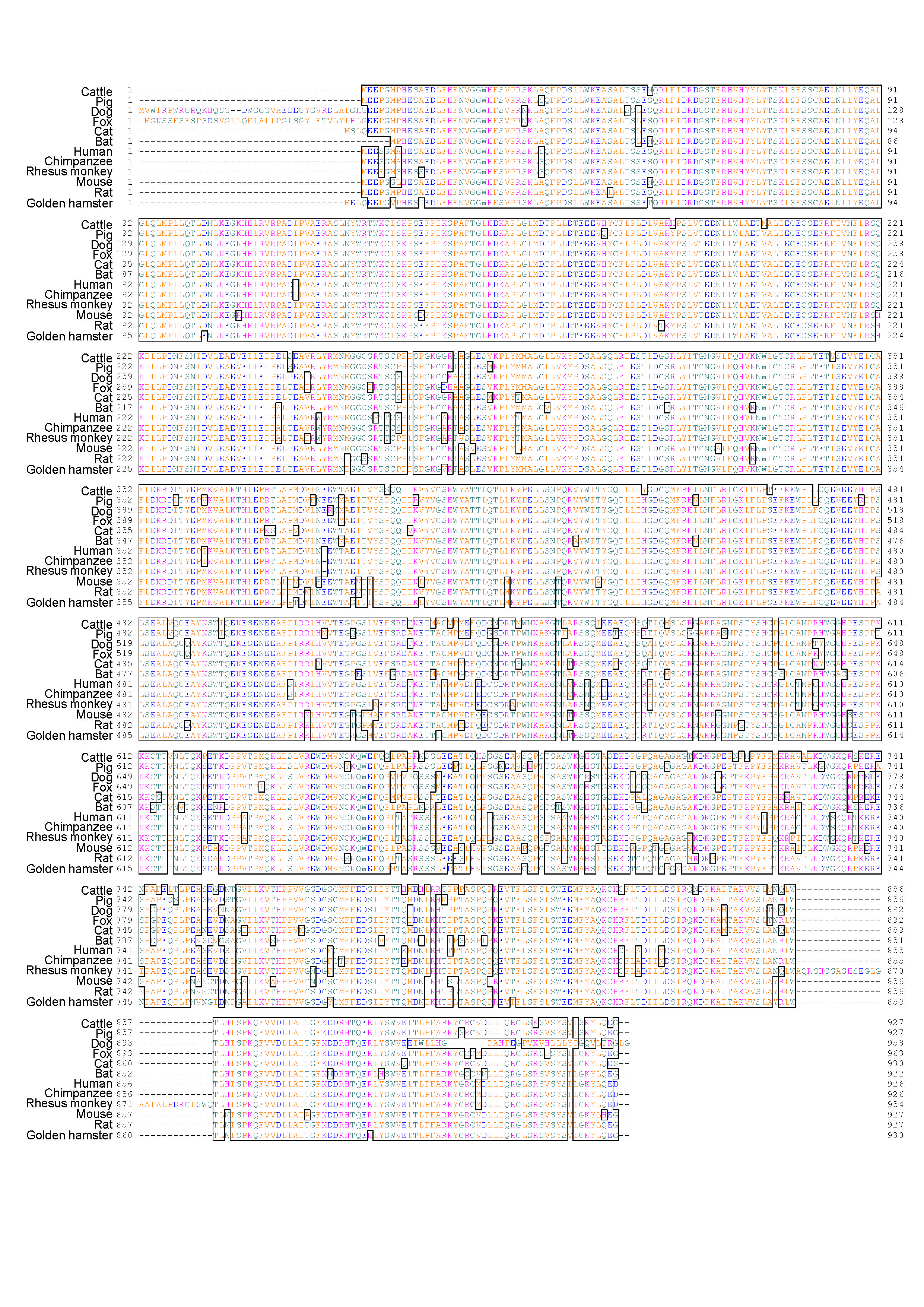

Supplement: S1 Fig — Prortein sequence comparison of KCTD19 in cattle (NP_001098862.1), pig (XP_003126977.2), dog (XP_022275030.1), fox (XP_025867456.1), cat (XP_023101865.1), bat (XP_027998908.1), human (NP_001094385.1), chimpanzee (XP_523391.2), rhesus monkey (XP_014981866.1), mouse (NP_808459.1), rat (NP_001292128.1), and golden hamster (XP_021086458.1). (TIF) [file pgen.1009412.s001.tif]

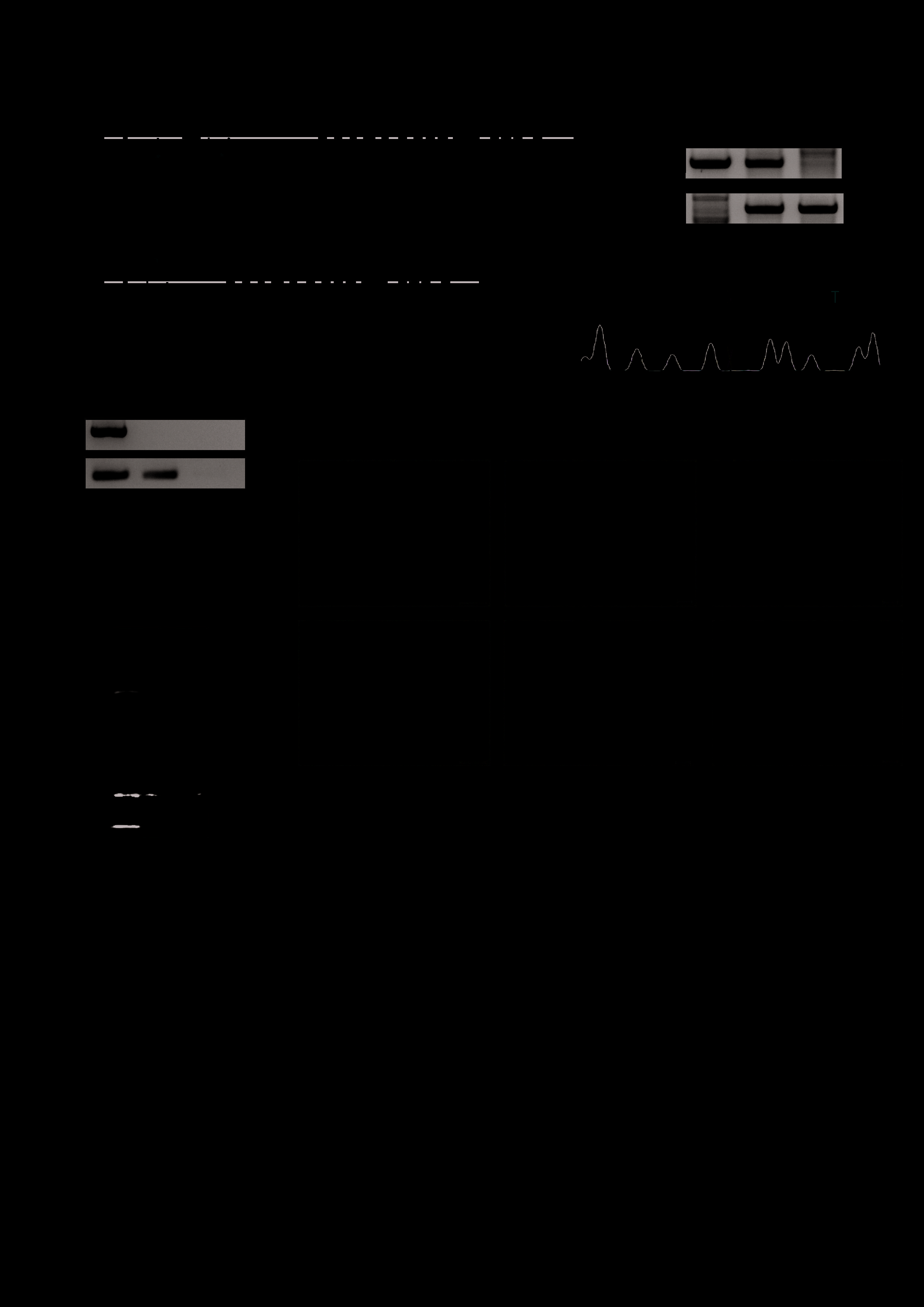

Supplement: S2 Fig — (A) Gene map of Kctd19. Black and white boxes indicate coding and non-coding regions, respectively. Black arrows and arrowheads indicate primers for genotyping and gRNAs for genome editing, respectively. (B) An example of genotyping PCR with two primer sets shown in S2A. (C) DNA sequencing verifies the deletion. (D) RT-PCR using testis cDNA obtained from WT and ΔBTB/ΔBTB mice. Actb was used as a loading control. (E) Immunoblotting using testis lysates obtained from WT, del/del, and ΔBTB/ΔBTB mice. (F) PAS staining of seminiferous tubules of adult mice. The seminiferous epithelium cycle was determined by germ cell position and nuclear morphology. (TIF) [file pgen.1009412.s002.tif]

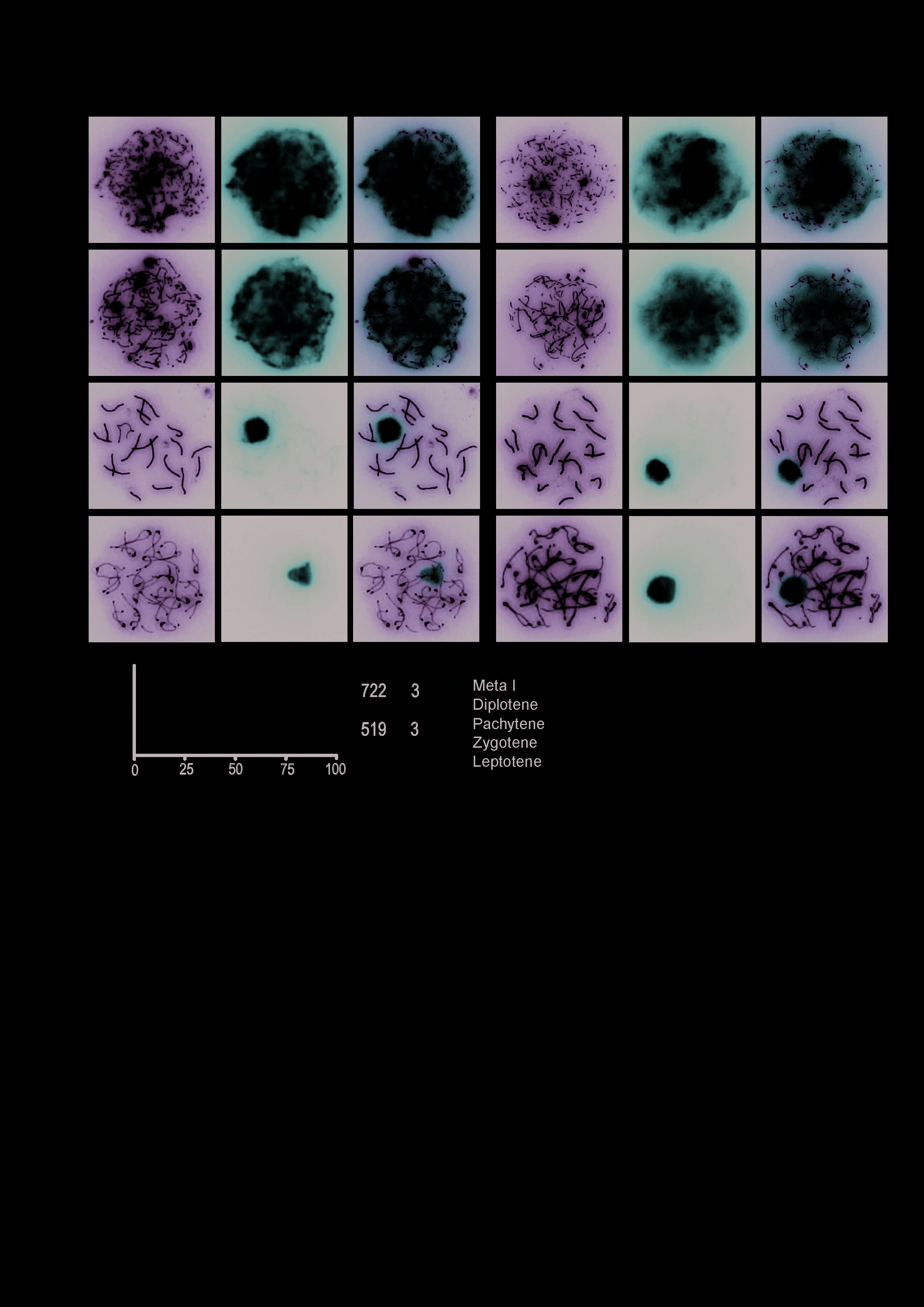

Supplement: S3 Fig — (A) Immunostaining of spread nuclei from prophase spermatocytes collected from juvenile mice (PND20). (B) The percentage of each meiotic prophase stage present is determined by immunostained spread nuclei samples. (TIF) [file pgen.1009412.s003.tif]

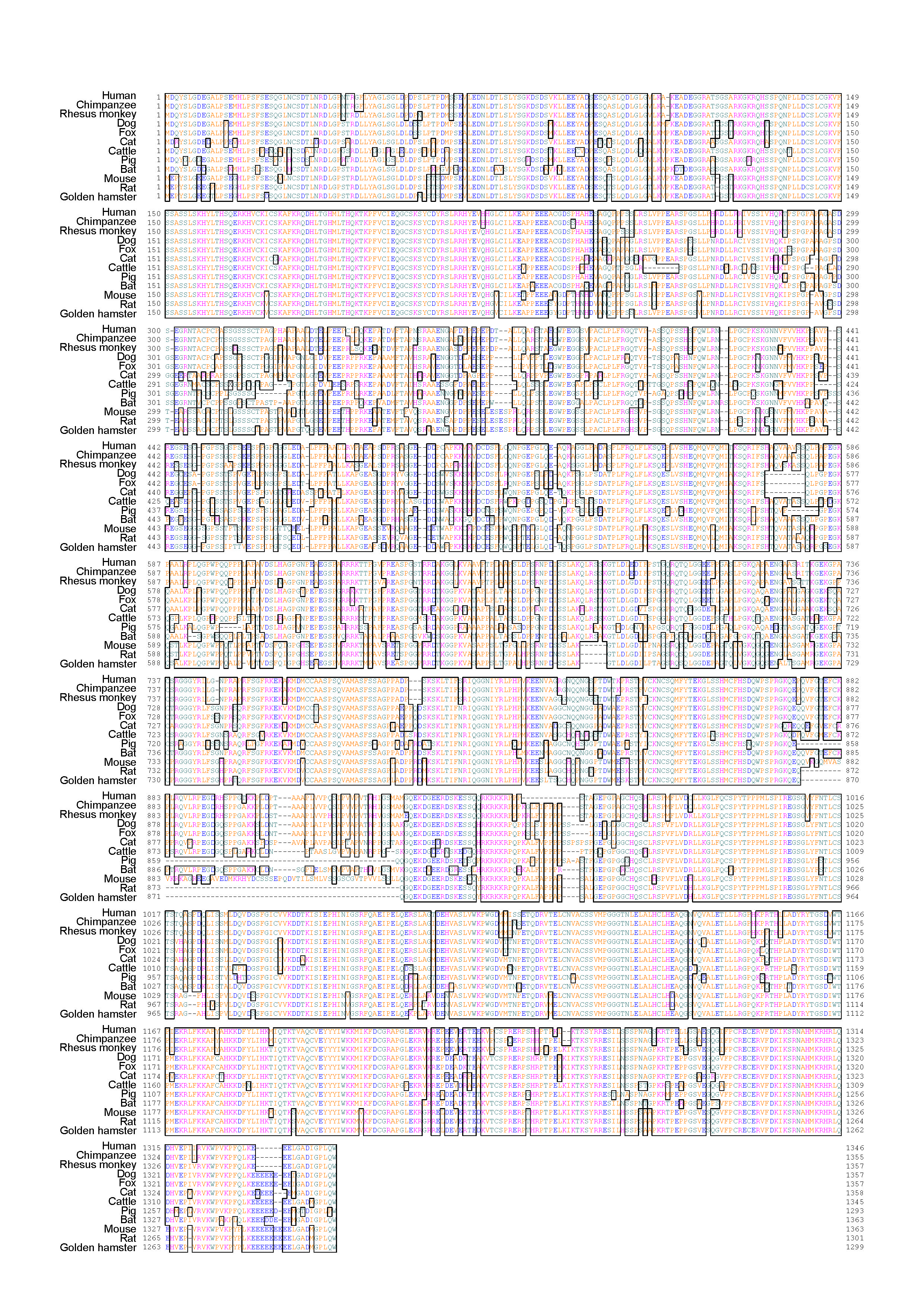

Supplement: S4 Fig — Prortein sequence comparison of ZFP541 proteins from various mammals: cattle (XP_015313711.2), pig (XP_020950303.1), dog (XP_005616437.1), fox (XP_025869832.1), cat (XP_023100994.1), bat (XP_008152641.1), human (NP_001264004.1), chimpanzee (XP_016791837.1), rhesus monkey (XP_014979842.2), mouse (NP_001092747.1), rat (NP_001100928.2), and golden hamster (XP_021078928.1). (TIF) [file pgen.1009412.s004.tif]

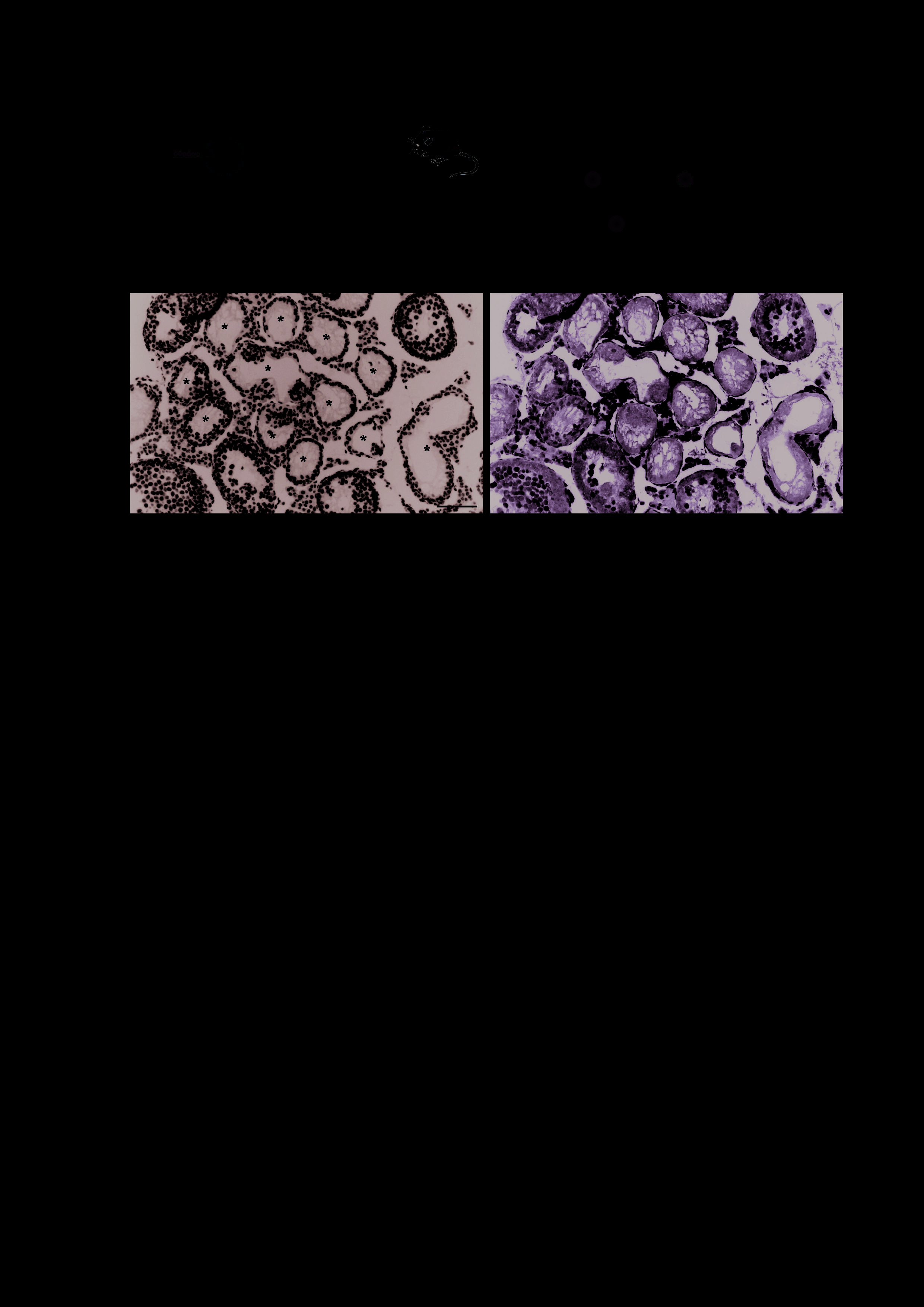

Supplement: S5 Fig — (A) Schematic of XY/XX chimeric mice production. XX prospermatogonia are eliminated around PND2. (B) Testis sections from chimeric mice. ES cell-derived cells were labeled with GFP fluorescence. Asterisk indicates depleted tubules. (TIF) [file pgen.1009412.s005.tif]
